# Scaffold-based 3D cellular models mimicking the heterogeneity of osteosarcoma stem cell niche

**DOI:** 10.1038/s41598-020-79448-y

**Published:** 2020-12-18

**Authors:** Giada Bassi, Silvia Panseri, Samuele Maria Dozio, Monica Sandri, Elisabetta Campodoni, Massimiliano Dapporto, Simone Sprio, Anna Tampieri, Monica Montesi

**Affiliations:** CNR-ISTEC, Institute of Science and Technology for Ceramics, National Research Council of Italy, 48018 Faenza, RA Italy

**Keywords:** Bone cancer, Cancer models, Cancer stem cells, Ceramics, Biomaterials, Bioinspired materials, Biomineralization

## Abstract

The failure of the osteosarcoma conventional therapies leads to the growing need for novel therapeutic strategies. The lack of specificity for the Cancer Stem Cells (CSCs) population has been recently identified as the main limitation in the current therapies. Moreover, the traditional two-dimensional (2D) in vitro models, employed in the drug testing and screening as well as in the study of cell and molecular biology, are affected by a poor in vitro-in vivo translation ability. To overcome these limitations, this work provides two tumour engineering approaches as new tools to address osteosarcoma and improve therapy outcomes. In detail, two different hydroxyapatite-based bone-mimicking scaffolds were used to recapitulate aspects of the in vivo tumour microenvironment, focusing on CSCs niche. The biological performance of human osteosarcoma cell lines (MG63 and SAOS-2) and enriched-CSCs were deeply analysed in these complex cell culture models. The results highlight the fundamental role of the tumour microenvironment proving the mimicry of osteosarcoma stem cell niche by the use of CSCs together with the biomimetic scaffolds, compared to conventional 2D culture systems. These advanced 3D cell culture in vitro tumour models could improve the predictivity of preclinical studies and strongly enhance the clinical translation.

## Introduction

Osteosarcoma is the most common primary malignant tumour of the bone^1^, frequently presenting in young people between the ages of 10–14 years and in adults over 65 years^2^. Typically, patients with osteosarcoma are subjected to a combination of surgery, radiotherapy and chemotherapy^3^, which together can lead to a survival rate improvement of another 5 years in patients with localised tumour^2,4–7^. However, while overall survival rate of non-metastatic tumour is about 60–70%, it remains less than 20% for patients with metastasis^8^. Moreover, tumour recurrences occur in 30–40% of non-metastatic patients^9^. Unfortunately, this scenario remained unchanged over the last 40 years, underlying the need of novel therapeutic strategies^10,11^. The therapeutic failure against osteosarcoma is mainly due to two reasons: (1) the lack of specificity for Cancer Stem Cells (CSCs) and (2) the absence of 3D microenvironment models that recapitulate the tumour complexity. Recent data have confirmed that osteosarcoma contains a distinct and defined cell population of CSCs, exhibiting stem-like phenotype with spherical colonies forming ability, called sarcospheres^12^, characterized by critical properties of invasiveness, migration and drug resistance^13,14^, suggesting their involvement in tumour progression, metastasis and recurrences frequently observed in osteosarcoma patients^15^. CSCs reside in an anatomically distinct and defined region inside the tumour microenvironment, called niche^16,17^, which controls CSC’s fate through the mutual feedback between the different cells and the extracellular matrix (ECM)^18–21^.

The second cause of the therapy failure is the lack of predictive in vitro models of the in vivo physio-pathological situation. Although many new tumour drug candidates seemed to be promising in in vitro screenings, they did not show any efficacy during in vivo studies^22^. This low translational ability can be attributed to the poor reliability of in vitro 2D standard models (i.e. plastic and glass surfaces)^23^. Although they are commonly used due to their controllability, simplicity, replicability and cheapness^24,25^, they do not reproduce the human disease complexity like the physico-chemical and mechanical tissue properties, the inter- and intra-tumour heterogeneity, the drug penetration through the tissue, the interaction between tumour cells and the stroma cells and the CSCs niche^1,20,21,26,27^. Recently, to overcome this great limitation, a significant effort has been made to clarify the dynamic interaction between the two players, cells and ECM, leading to the Tissue Engineering’s evolution into “Tumour Engineering”, with the aim to engineer the 3D tumour microenvironment and better elucidate several biological events and discover disruptive therapies^1,28^.

In line with this challenge, we proposed two different 3D cancer models that recapitulate the native osteosarcoma heterogeneous CSCs niche. Our approach involved the combination of 3D scaffolds able to mimic the bone extracellular matrix, providing a structural support, specific physico-chemical and biomechanical stimuli to tumour cells^29,30^, together with enriched-CSCs obtained by sarcosphere-forming culture starting from osteosarcoma cell lines (MG63 and SAOS-2)^31^. In detail a biomimetic hybrid composite scaffolds obtained by a biomineralization process involving the direct nucleation of Mg-doped hydroxyapatite (MgHA) on self-assembling collagen fibres (MgHA/Coll)^32,33^ and porous hydroxyapatite scaffolds (HA) produced by direct foaming process^34^, were used. An extensive characterization of morphology and gene expression profile of osteosarcoma cell lines and enriched-CSCs cultured in standard 2D conditions *versus* the proposed 3D culture systems has been performed. The results showed how the CSCs maintained more their stemness features when cultured in both MgHA/Coll and HA biomaterials compared to 2D model. The proposed biomimetic scaffolds, recapitulating the stem cell niche composition and architecture of native tissue, represent promising 3D tumour models that could replace the standard in vitro screening models closing the gap between the drug discovery and the clinical translation.

## Results

### Sarcospheres characterization

**Figure 1:**
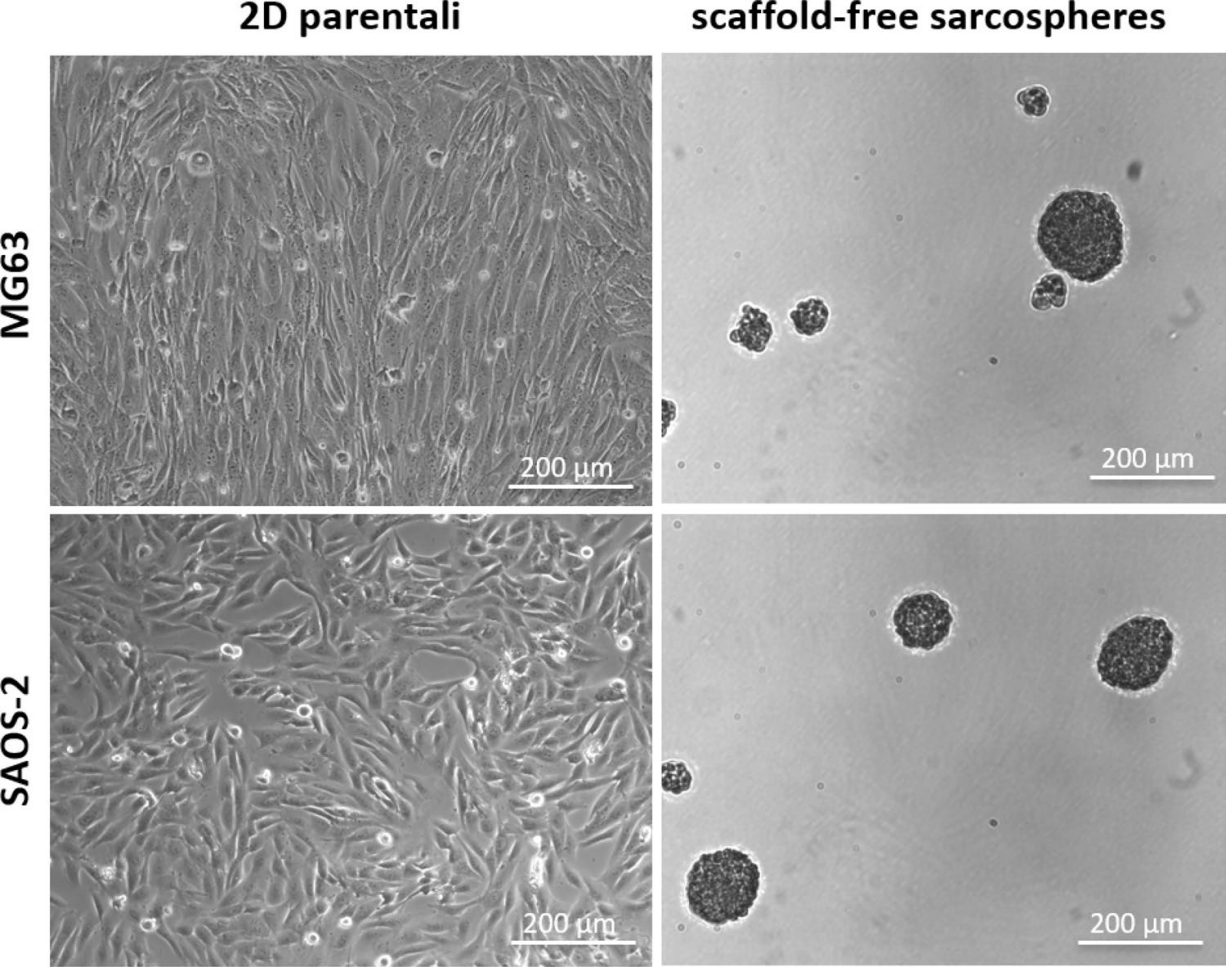
Qualitative morphological characterization of sarcospheres. Parental cells grown adherent to 2D standard support showing their typical morphologies. Stable floating sarcospheres.

MG63 and SAOS-2 cell lines were subjected to sarcosphere-forming culture, following the well-established methods for CSCs enrichment^35–37^. After 10 days of culture, the scaffold-free sarcospheres were characterized to confirm the successful CSCs’ enrichment. A qualitative morphological evaluation confirmed the successful formation of stable CSCs’ spheroids with diameter ≥ 50 µm (Fig. 1).

**Figure 2:**
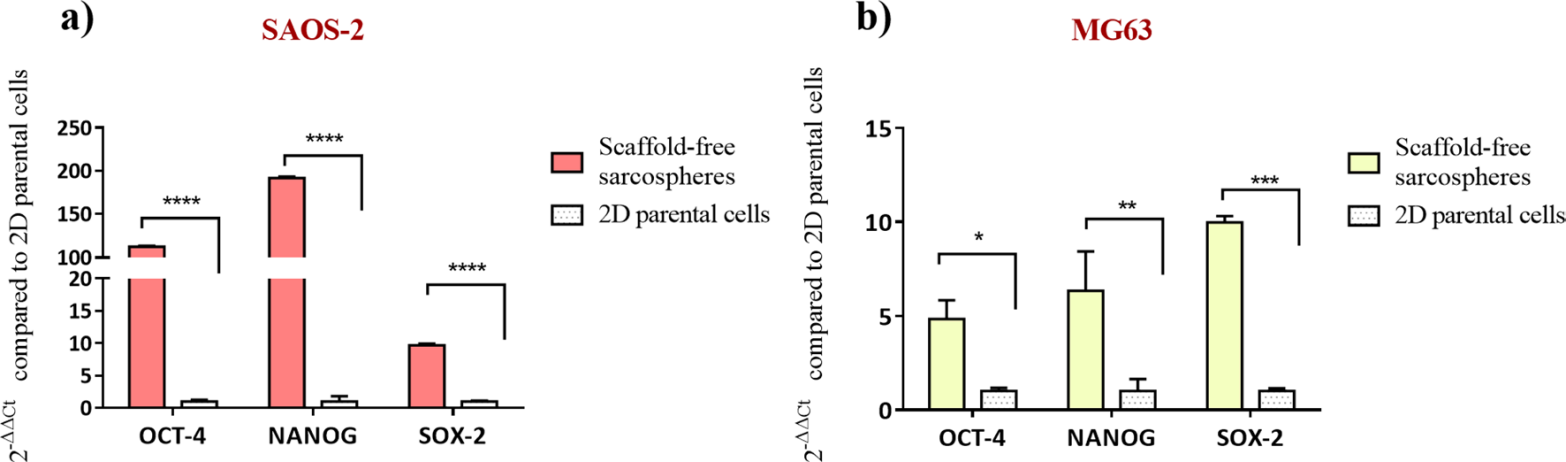
Gene expression analysis of scaffold-free sarcospheres. Relative quantification of scaffold-free sarcospheres gene expression of OCT-4, NANOG and SOX-2 in (**a**) SAOS-2 and (**b**) MG63 cell lines by qPCR. The graphs show the fold change expression of the genes relative to 2D parental cells (mean ± standard error; ****p value ≤ 0.0001; ***p value ≤ 0.001; **p value ≤ 0.01; *p value ≤ 0.05).

The relative quantification of OCT-4, NANOG and SOX-2 genes was performed in order to define the increasing expression of stemness genes in the enriched-CSCs of the scaffold-free sarcospheres compared to 2D parental cells. The results showed a statistically significant higher expression of the three stemness markers in both cell lines. Transcriptional factor OCT-4, NANOG and SOX-2 were statistically significant higher in SAOS-2 sarcospheres (*p* value ≤ 0.0001 for all genes) compared to 2D parental cells. MG63 sarcospheres showed a statistically significant higher expression of OCT-4 (*p* value ≤ 0.05), NANOG (*p* value ≤ 0.01) and SOX-2 (*p* value ≤ 0.001), compared to the parental control (Fig. 2).

### In vitro 3D osteosarcoma models: analysis of cell-biomaterial interaction

#### Cell morphology analysis

**Figure 3:**
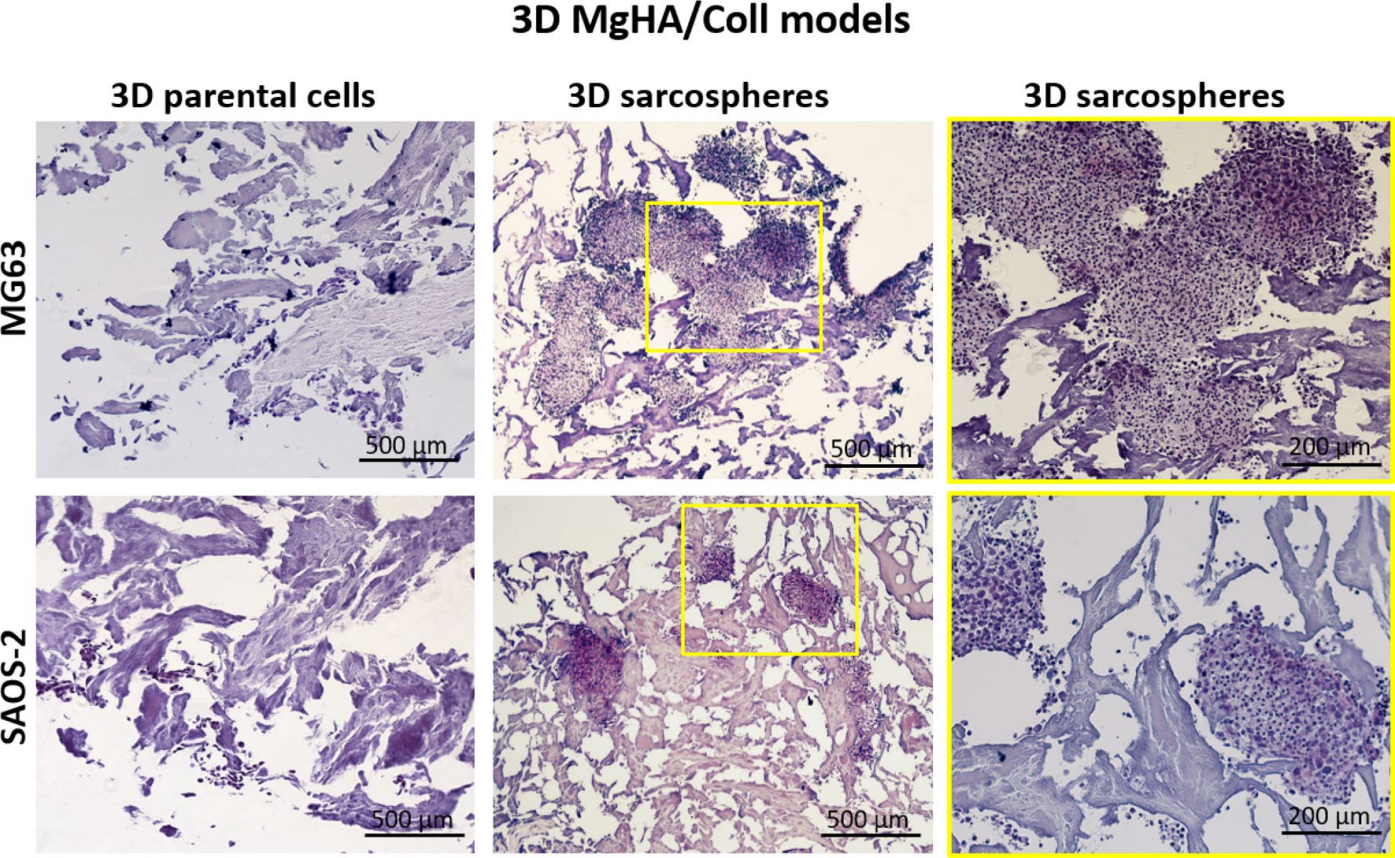
Histological analysis of the 3D MgHA/Coll scaffolds with both MG63 and SAOS-2 cells. The images highlight the morphological features and the interaction behaviour of the sarcospheres and parental cells with the MgHA/Coll material. On the right, it is possible to observe an image enlargement of 200 µm of the spheroidal phenotype of the sarcospheres.

After 10 days of culture, the cell-biomaterial interaction was analysed looking at the morphology of sarcospheres and the parental cells, respectively, grown in MgHA/Coll and HA 3D scaffolds. The H&E staining of MgHA/Coll sample showed no differences between MG63 and SAOS-2 cells. In detail sarcospheres preserved their round-shape morphology, respect to parental cells, although they were well-embedded into the scaffold matrix (Fig. 3).

**Figure 4:**
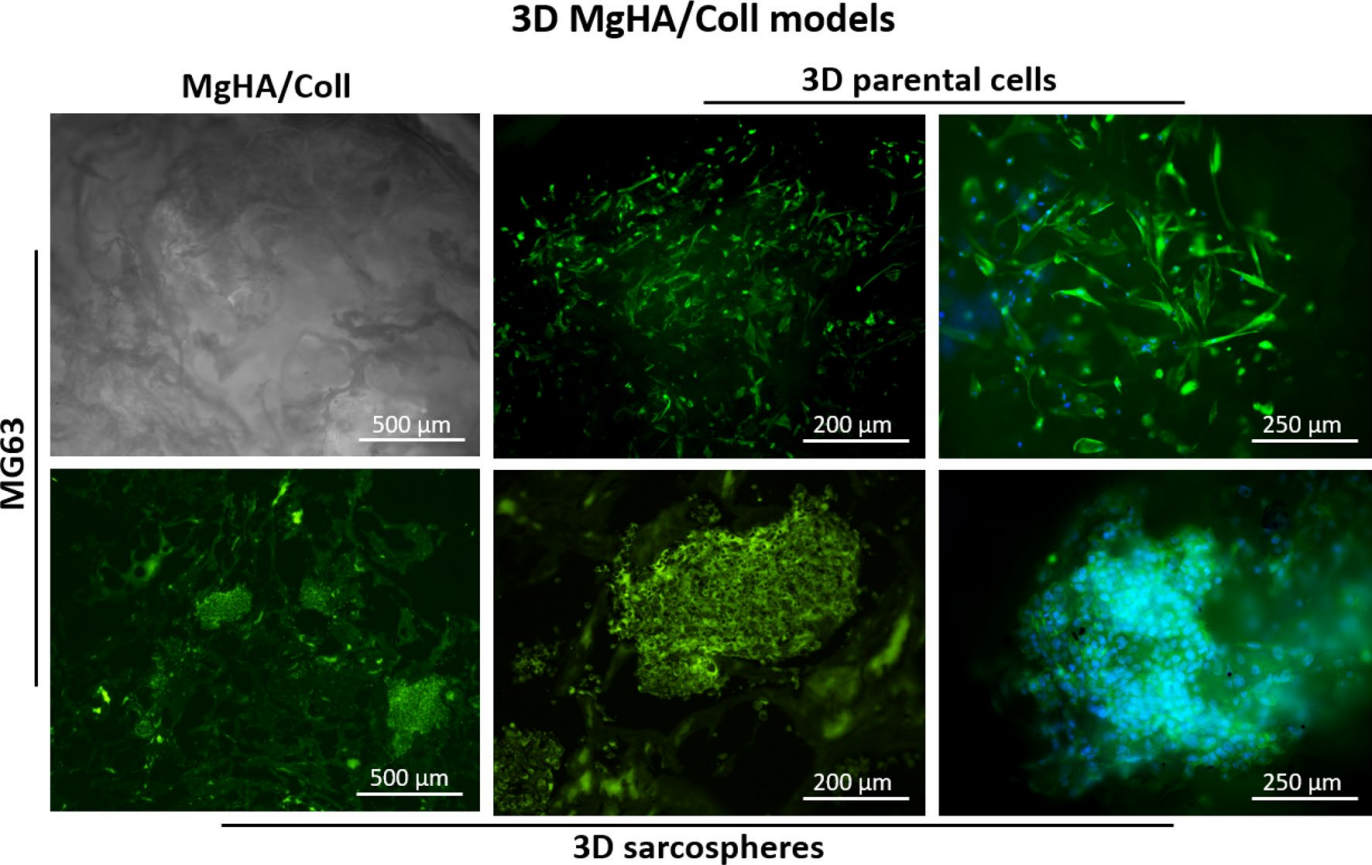
Fluorescence analysis of the 3D MgHA/Coll model of MG63 sarcospheres and parental cells. The top left figure is a representative image of the MgHA/Coll material. The panel shows the two cell phenotypes and their interaction with the hybrid material; cell nuclei in blue (DAPI) and F-actin filaments in green (Phalloidin).

The actin filaments fluorescence analysis also confirmed an excellent cell-ECM interaction of both cell phenotypes with no significant differences. Figure 4 reported a representative panel of MG63 parental cells and MG63 sarcospheres grown in MgHA/Coll. The images highlight the complex interconnected structures of the scaffold and the maintenance of the cell-specific phenotypes morphology (Fig. 4).

**Figure 5:**
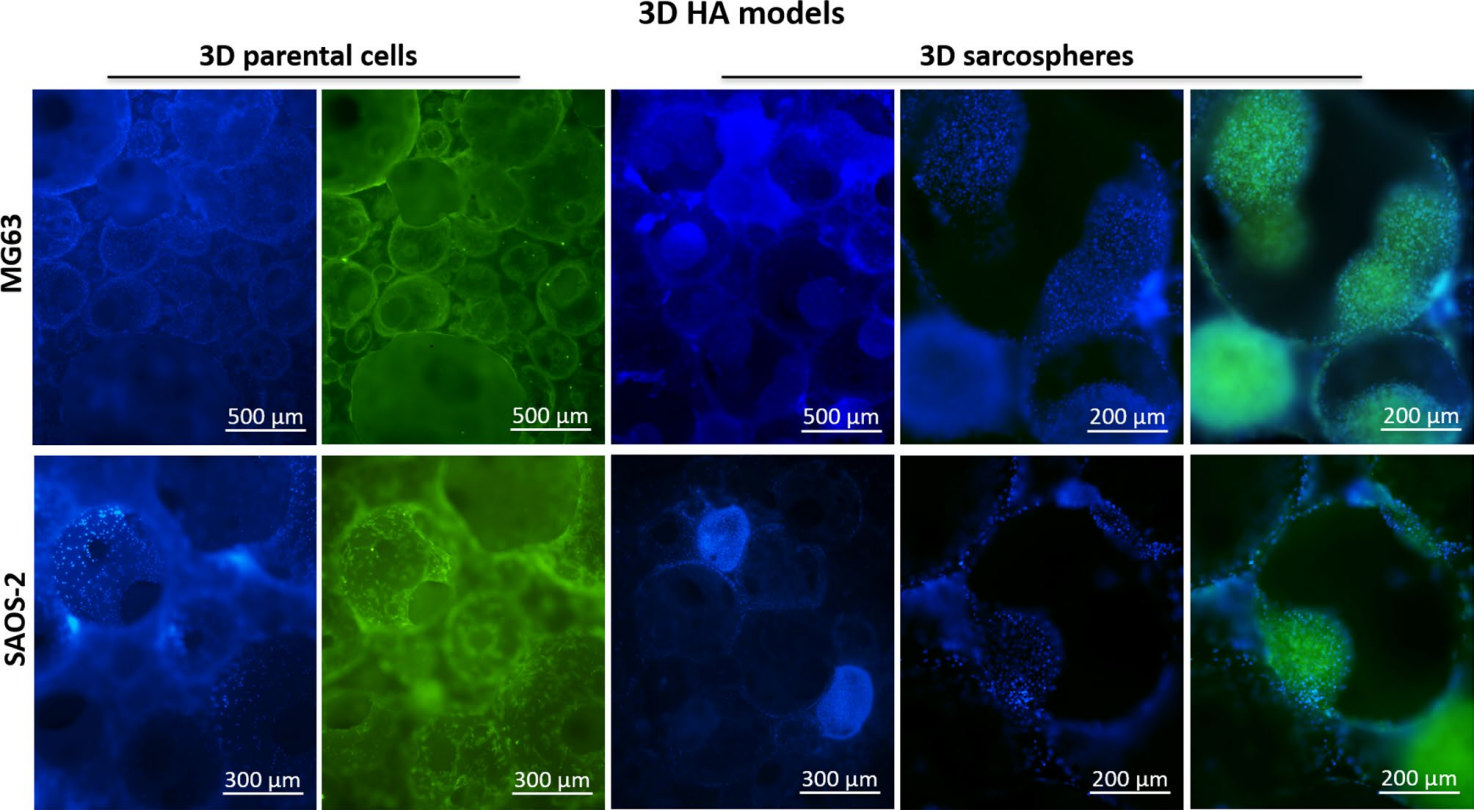
Fluorescence analysis of the 3D HA model of MG63 and SAOS-2 sarcospheres and parental cells. The images highlight the two cell phenotypes and their interaction with HA material; cell nuclei in blue (DAPI) and F-actin filaments in green (Phalloidin).

The fluorescence analysis was performed also with the 3D HA model and it showed a peculiar round-shaped porous morphology of the HA scaffold (Fig. 5). The parental cells entirely colonized the biomaterial showing the typical adhesion morphology, while sarcospheres colonized the pores of the HA scaffolds preserving their spheroidal phenotype without differences between the MG63 and SAOS-2 (Fig. 5).

**Figure 6:**
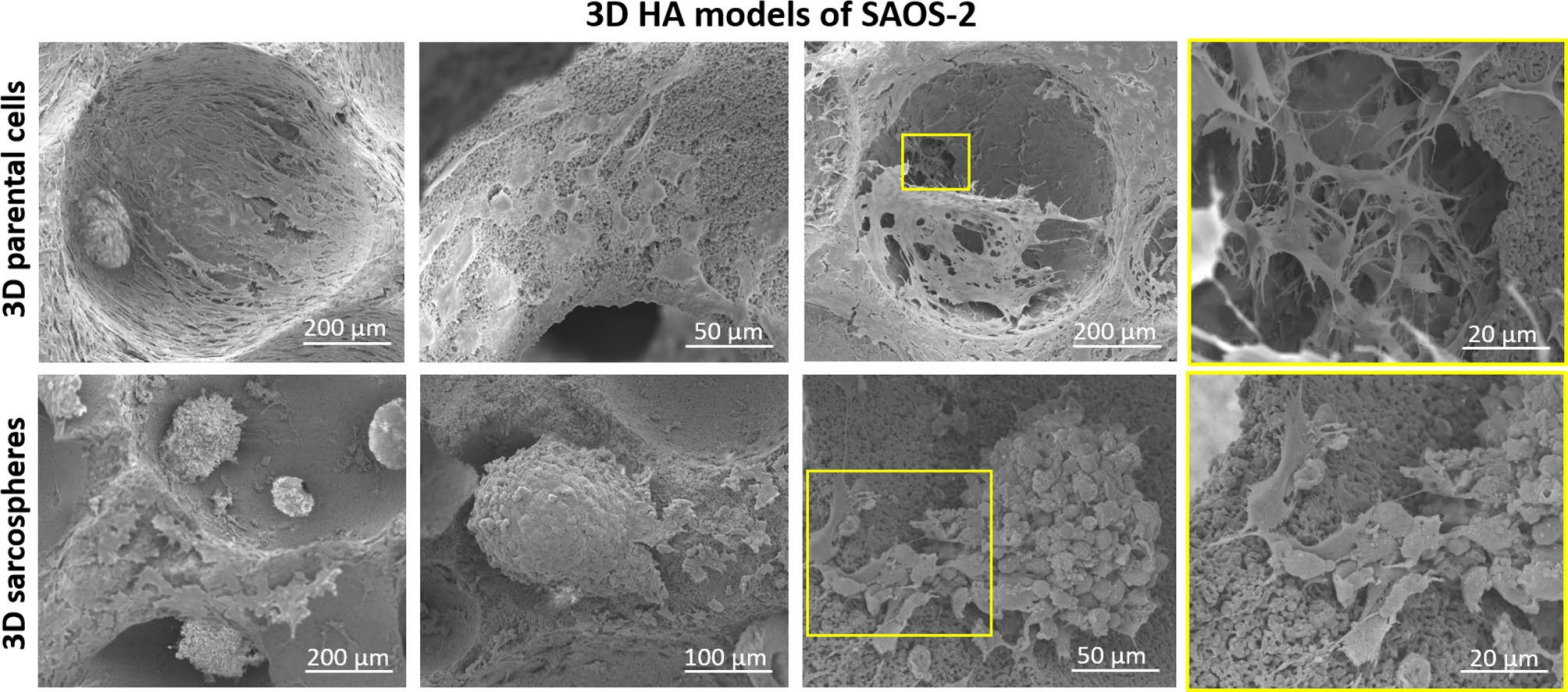
SEM analysis of the 3D HA model of SAOS-2 cells. The figure highlights phenotypic differences between sarcospheres and parental cells and interaction abilities of the cells toward HA scaffold.

Likewise, SEM analysis confirmed the obtained results, providing a more detailed cell morphology evaluation of the 3D HA models (Fig. 6). Since no differences were observed in the morphology of the two cell lines, it has been reported a representative panel of SEM images of SAOS-2 cells. Parental cells were well spread over the biomaterial surface, with cytoplasmic extensions interconnecting cell-to-cell and cell-to-biomaterial nanostructured surface. Moreover, the images showed the typical spheroidal phenotype of SAOS-2 sarcospheres, highlighting a tight sphere-edge interaction with the HA scaffold (Fig. 6).

#### Gene expression and immunofluorescence analysis

**Figure 7:**
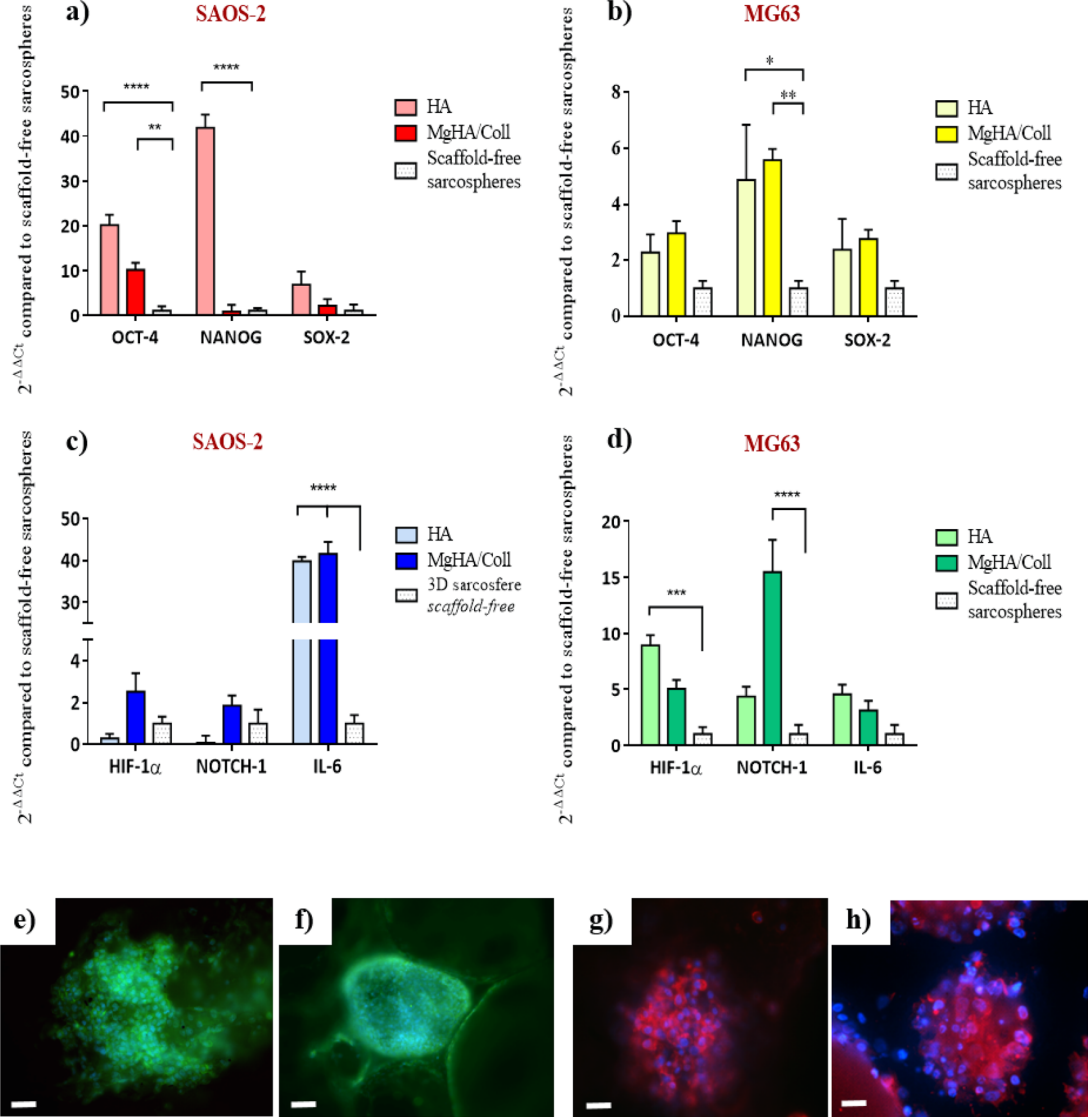
Gene expression and protein immunofluorescence analysis of 3D sarcospheres scaffold-based models. Relative quantification of OCT-4, NANOG and SOX-2, stemness marker genes, SAOS-2 (**a**) and MG63 (**b**). Relative quantification of HIF-1a, NOTCH-1 and IL-6, CSCs niche-related genes, SAOS-2 (**c**) and MG63 (**d**). The graphs show the fold change expression of the genes relative to the scaffold-free sarcospheres at day 10 of culture (mean ± standard error; *p value ≤ 0.05; **p value ≤ 0.01; ***p value ≤ 0.001; ****p value ≤ 0.0001;). The panel shows a representative image of OCT-4 immunolocalization in SAOS-2 sarcorpheres MgHA/Coll model (**e**) and in HA model (**f**) scale bar 50 µm; SOX-2 immunolocalization in MG63 sarcorpheres MgHA/Coll model (**g**) and in HA model (**h**) scale bar 25 µm. Blu: cell nuclei; green: OCT-4; red: SOX-2.

After 10 days of culture, the expression profile of genes involved in the stemness and CSCs/niche communication was relative quantified by qPCR (Fig. 7). OCT-4, NANOG and SOX-2 stemness marker genes were analysed to determine sarcospheres stem phenotype grown in 3D biomimetic scaffolds compared to the expression rate in the scaffold-free sarcospheres (Fig. 7a). The results showed that 3D sarcospheres scaffold-based models induced a significant up-regulation of NANOG in 3D HA model for SAOS-2 and MG63 cell line (~ 40.9 and 4.8-fold change, respectively) (*p* value ≤ 0.0001 and ≤ 0.05, respectively) and in 3D MgHA/Coll model for MG63 cell line (~ 4.6-fold change) (*p* value ≤ 0.01), compared to scaffold-free model. SAOS-2 sarcospheres also showed a significant increase of OCT-4 expression in both 3D HA (~ 19.2-fold change) (*p* value ≤ 0.0001) and MgHA/Coll model (~ 9.1-fold change) (*p* value ≤ 0.01). Finally, both cell lines showed a trend of increasing expression of SOX-2 in 3D scaffold-based models, even no statistically significant difference was detected (Fig. 7a).

Moreover, relative quantification of NOTCH-1, HIF-1α and IL-6, the genes related to CSCs niche interaction, was carried out. The Fig. 7b showed higher expression of signalling genes in the 3D scaffold-based sarcospheres compared to those without scaffolds. In detail, MG63 sarcospheres proved a significantly higher expression of NOTCH-1 (~ 14.5-fold change) (*p* value ≤ 0.0001), and HIF-1α (*p* value ≤ 0.001) when grown in both 3D MgHA/Coll (~ 4.0-fold change) and HA model (~ 7.9-fold change), compared to scaffold-free sarcospheres. In the same way, SAOS-2 cell line showed a significant up-regulation of IL-6 in 3D sarcospheres with both material types (~ 38.8-fold change and ~ 40.6-fold change in 3D HA and MgHA/Coll model, respectively) with a *p* value ≤ 0.0001 (Fig. 7b). The qualitative evaluation of OCT-4 and SOX-2, by immunofluorescence straining, confirmed the expression of these stemness markers. No differences have been observed in OCT-4 and SOX-2 protein expression among the 3D scaffold-based models, therefore one representative image for each model has been reported (Fig. 7e,f,g,h).

## Discussion

The incidence of osteosarcoma recurrence and metastasis due to the failure of the conventional therapies is strictly related to the critical role of CSCs, presented in several tumours, that exhibit stem-like phenotype and high drug resistance suggesting their involvement in tumour invasiveness and metastasis frequently observed in osteosarcoma patients^15^. Moreover, the lack of predictive in vitro models of the real physio-pathological scenario leads to a poor clinical translation of many apparently promising drugs candidates but also to fail in deciphering the biological events. In fact the 2D culture systems, used in preclinical drug-screening and in the study of cells and molecular behaviours, represent standard models but with plastic and/or glass surfaces very different from the osteosarcoma environment^1,23,38,39^.

The present study proposes a Tumour Engineering approach offering a radical change in the osteosarcoma in vitro studies through the development of advanced and alternative 3D cell culture in vitro models, able to mimic the *in viv*o tumour microenvironment. We studied the CSCs behaviour in two 3D biomimetic scaffolds (MgHA/Coll and HA) and we proved how these bone-like biomaterials provide a more mimetic tumour microenvironment, in terms of nanostructure and physico-chemical features, compared to the 2D conventional culture systems. The MgHA/Coll and HA scaffolds were designed for bone regeneration and previously fully characterized by our group^32–34,40–43^. They are biocompatible, bioresorbable and they display osteogenic properties, mainly due to the presence of biomimetic HA^44^. The hybrid MgHA/Coll scaffold is obtained by a bio-inspired mineralization process reproducing the cascade of phenomena occurring in vivo during the formation of new bone tissue^32,33^. In particular the process, carefully regulated by pH and temperature control, activates physico-chemical and structural control mechanisms yielding the supramolecular assembling of Type I collagen fibrils and, at the same time, the heterogeneous nucleation of nanocrystals of MgHA , so as to obtain a fibrous hybrid construct, stabilized by DHT crosslinking process, closely mimicking the woven bone tissue^41,45,46^ (Fig. 4). The HA scaffold is a sintered porous ceramic body obtained by a direct foaming process yielding open and highly interconnected macro/micro-porosity, thanks to the controlled formation of air bubbles incorporated into the hydroxyapatite ceramic slurry, obtained as the precursor of the final device ^28,34,42,43,47–51^. Both the biomimetic scaffolds show a nanostructural organisation that, along with their peculiar chemistry, enhances the cell adhesion, migration and subsequent cell colonisation.

It has been well demonstrated that tumour spheroids provided high performance and a more accurate in vitro model for biological study of tumour and CSCs-tumour niche behaviour^52^, assuming the role of excellent candidates for the identification of novel therapeutic targets and the evaluation of sensitivity to chemotherapeutic agents in order to eradicate the CSCs niche^53^. With the aim to ascertain the efficiency of the CSCs enrichment method, both qualitative and quantitative analysis were performed to characterize the sarcospheres of both MG63 and SAOS-2 cell lines, by using the 2D parental cells model as experimental control. The results provide evidences of the formation of stable sarcospheres showing in vitro spheroidal phenotype and an higher mRNA level of stemness genes OCT-4, SOX-2 and NANOG^36,54^ compared to 2D parental cells (Figs. 1, 2), confirming the successful CSCs enrichment by sarcospheres formation^55,56^. To achieve a more closed tumour biomimesis the sarcospheres were cultured in the 3D scaffolds. The morphological analysis confirmed the favourable environment for tumour cells. In fact the high biomimicry and bioactivity of both scaffolds did not induce the loss of the spheroidal phenotype of the sarcospheres, allowing their anchor within the HA pores and on MgHA/Coll fibres preserving their primary marker for CSCs identification^57^ without differences between the two cell lines (Figs. 3, 4, 5, 6).

It is well established that the presence of a bioactive 3D environment provides a higher stimulation of genes compared to a simple inducing medium, assuming high relevance for the reproduction of an in vitro more closely predictive osteosarcoma model^58,59^. In fact, many studies reported how the use of conventional 2D approaches failed to explain tumour cell biology, because they did not mimic real macrostructure, complexity (tumour-stroma interactions) and heterogeneity of the tumour microenvironment^28,60^. In the proposed 3D models the significant upregulation of genes OCT-4, NANOG and SOX-2, typically used as stemness markers due to their essentiality in preserving the pluripotency and the self-renewal property of cancer stem cells^36,61–64^ , demonstrated that the presence of 3D biomimetic scaffold, reproducing the nanostructure and physico-chemical features of native environment, induces the higher stem phenotype in 3D sarcospheres compared to those grown without scaffolds (Fig. 7a,b). The stemness was also confirmed by the detection of SOX-2 and OCT-4 proteins expressions, revealed by immunofluorescence analysis, in all the 3D scaffold-based models (Fig. 7e,f,g,h).

Moreover, it is well-known the significant contribute of ECM mechanical properties on tumour progression^65^, with particular attention to the stiffness of the material affecting tumour and stem cells fate and providing a tissue-specific microenvironment that plays a critical role in tumour development^66,67^. The two biomaterials used in this study exhibited significantly different , mechanical behaviours (i.e. Young’s Modulus) giving the chance to compare the effect of high-stiffness HA scaffolds (1.8 ± 0.2 GPa) with low-stiffness MgHA/Coll scaffolds (30.93 ± 6.14 kPa) on sarcospheres behaviour^32,34^. Some studies reported that CSCs of osteosarcoma interacted optimally with 50–55 kPa substrate stiffness^68^. In this respect, our results showed a higher expression of CSCs niche related-genes in 3D MgHA/Coll scaffold. In addition, it has been shown that in vitro invasive cancer cells have a higher affinity for matrices expressing the Type I Collagen^69^. At the same time, some studies reported that increasing ECM stiffness induces malignant phenotypes contributing to cancer progression and metastasis^70,71^, supporting the observed high expression levels of stemness genes in SAOS-2 sarcospheres in the 3D HA scaffold.

In order to clarify the role of the mimetic biomaterials on the CSCs niche-mediated stimuli, the expression of NOTCH-1, HIF-1α and IL-6 was analysed. Although they have been observed different gene expression profiles related to the different scaffolds and cell types, overall a significant up-regulation of some of those genes was observed in 3D sarcospheres scaffold-based compared to those grown without scaffolds, confirming the greater mimesis of in vivo tumour microenvironment (Fig. 7c,d). In fact, these genes are typically involved in the signalling between CSCs and the tumour stem niche, developing a complex intercellular communication network regulating stemness and CSCs fate^72^. The signals are typically deregulated in osteosarcoma and they are involved in self-renewal, differentiation, drug resistance and metastatic potential regulation of CSCs^73^. In our study NOTCH-1 and HIF-1α were higher expressed in MG63 sarcospheres grown in 3D MgHA/Coll and HA scaffold, respectively, compared to the scaffold-free model, confirming the active role of 3D mimetic environment on conservation of CSC phenotype^74–76^. Previous studies reported an increased level of IL-6 in the serum of patients with osteosarcoma^77^ and highlighted how IL-6, together with other cytokines (e.g. IL-8^78,79^ and CXCL12^80^), promotes immunosuppressive function, increasing chemoresistance^81,82^ and local and systemic tumour aggressiveness^83^. The IL-6 increase was observed also in our models where a statistically significant up-regulation in 3D sarcospheres of SAOS-2 cell line with both HA and MgHA/Coll biomaterial was reported (Fig. 7c).

Although the molecular analysis showed slight variances attributable to the intrinsic biological differences of the two osteosarcoma cell lines^84^ and to the specific biomimetic scaffold features^49^, the overall results confirmed that the use of the 3D scaffolds, together with sarcospheres implementation, improved the osteosarcoma stem cell niche microenvironment simulation, providing precise inputs supporting cell–cell and cell-ECM interactions and tumour signalling pathways in vitro*.* Also in vivo intratumoral variability can occur between tumour cells arising from the same mass, leading to the classification of different tumour cell subtypes that show a range of functional and morphological properties and a different molecular profile. Moreover, the extrinsic interactions between tumour cells and stromal microenvironments may be involved in the in vivo tumour heterogeneity as a crucial determinant of tumour malignancy^84,85^.

Although recognizing the limitations of our simplified 3D osteosarcoma models, they provided a more accurate starting point to understand the cellular and molecular mechanisms involved in cancer cells/biomatrix interactions, particularly in CSCs population. Most importantly, these models contribute to overcome the use of conventional 2D culture systems and to acquire overall awareness towards the implementation of 3D culture systems in our daily in vitro experiments and screenings.

## Methods

### Biomimetic scaffolds

The hybrid composite scaffold is composed of hydroxyapatite nanocrystals nucleated on self-assembled type I collagen fibres ((MgHA/Coll 60/40%) and is obtained by a biomineralization process already reported in previous papers^32,33,40^. Briefly, an acid aqueous suspension was prepared by dispersing 150 g of 1 wt.% collagen gel (equine tendon derived type I collagen, 1 wt.% in aqueous acetic buffered solution pH 3.5, Opocrin SpA, Italy) into phosphoric acid solution (2.41 g in 500 ml; H_3_PO_4_, 85 wt.%, Sigma Aldrich, USA) at room temperature, while a basic aqueous suspension was obtained by adding 0.35 g of magnesium chloride (MgCl_2_·6H_2_O, 99 wt.%, Sigma Aldrich, USA) into a calcium hydroxide suspension (2.71 g in 500 ml; Ca(OH)_2_, 95 wt.%, Sigma Aldrich, USA) at room temperature. Later, the acid suspension was dropped into the basic suspension at 25 °C under constant stirring conditions causing the heterogeneous nucleation of MgHA nanocrystals onto the collagen fibres simultaneously at their assembling, thus forming a hybrid MgHA/Coll hydrogel. After 2 h of maturation at 25 °C, the hydrogel was lyophilized by freezing at – 40 °C and drying at 25 °C for 48 h under constant 0.086 mbar vacuum conditions (5 Pa, LIO 3000 PLT, Italy). Finally, the scaffolds were crosslinked by Dehydrothermal Treatment (DHT) into an oven at 160 °C for 48 h under a pressure of 0.01 mbar^32,33^.

The porous hydroxyapatite scaffold (HA) was obtained by a previously reported direct foaming process^34^. Briefly, commercial HA powder (Riedel de Haen, Germany) was calcinated at 1000 °C for 5 h and dispersed in water with Dolapix CA (Zschimmer and Schwartz, Germany), following the weight ratio HA:H_2_O:dispersant = 73:23:4. After 30 min stirring at 400 rpm (Pulverisette 6, Fritsch, Germany) foaming agents were added to the suspension according to the HA powder amount, 2 wt.% of Olympicon A (Olympia Surfactants, Italy) and 0.7 wt.% of W53 (Zschimmer and Schwartz, Germany), followed by further 5 min stirring. The as-obtained foamed suspension was finally poured into paper moulds, dried for 2 days at room temperature and then sintered at 1250 °C for 1 hour^34^.

Both types of biomaterial (3.00 mm high and 8.00 mm of diameter) were washed and sterilized by performing 25 kGy γ-ray irradiation before the use.

### Cell culture

The Human Osteosarcoma SAOS-2 cell line purchased from American Type Culture Collection (ATCC hTB-85) were cultured in standard medium composed by McCoy’s 5A (Modified) Medium (Gibco) supplemented with 15% of Foetal Bovine Serum (FBS) and 1% of a mixture of Penicillin and Streptomycin (Pen/Strep) (100–100 μg/ml).

The Human Osteosarcoma MG63 cell line purchased from American Type Culture Collection (ATCC CRL-1427) were cultured in standard medium composed by Dulbecco’s Modified Eagle Medium/F-12 Nutrient Mixture (DMEM/F-12) with Glutamine (GlutaMAX) (Gibco), supplemented with 10% of FBS and 1% of Pen/Strep. Both cell lines were kept in an incubator at 37 °C and in 5% CO_2_ atmosphere conditions. Cells were detached from culture plastics by trypsinization and centrifuged. The cell number and viability were determined with Trypan Blue Dye exclusion test. These cultures are named 2D parental cells models. All cell handling procedures were performed under laminar flow hood and in sterility conditions. The culture systems were incubated at 37 °C and in 5% CO_2_ atmosphere conditions.

### Sarcosphere-forming culture

**Figure 8:**
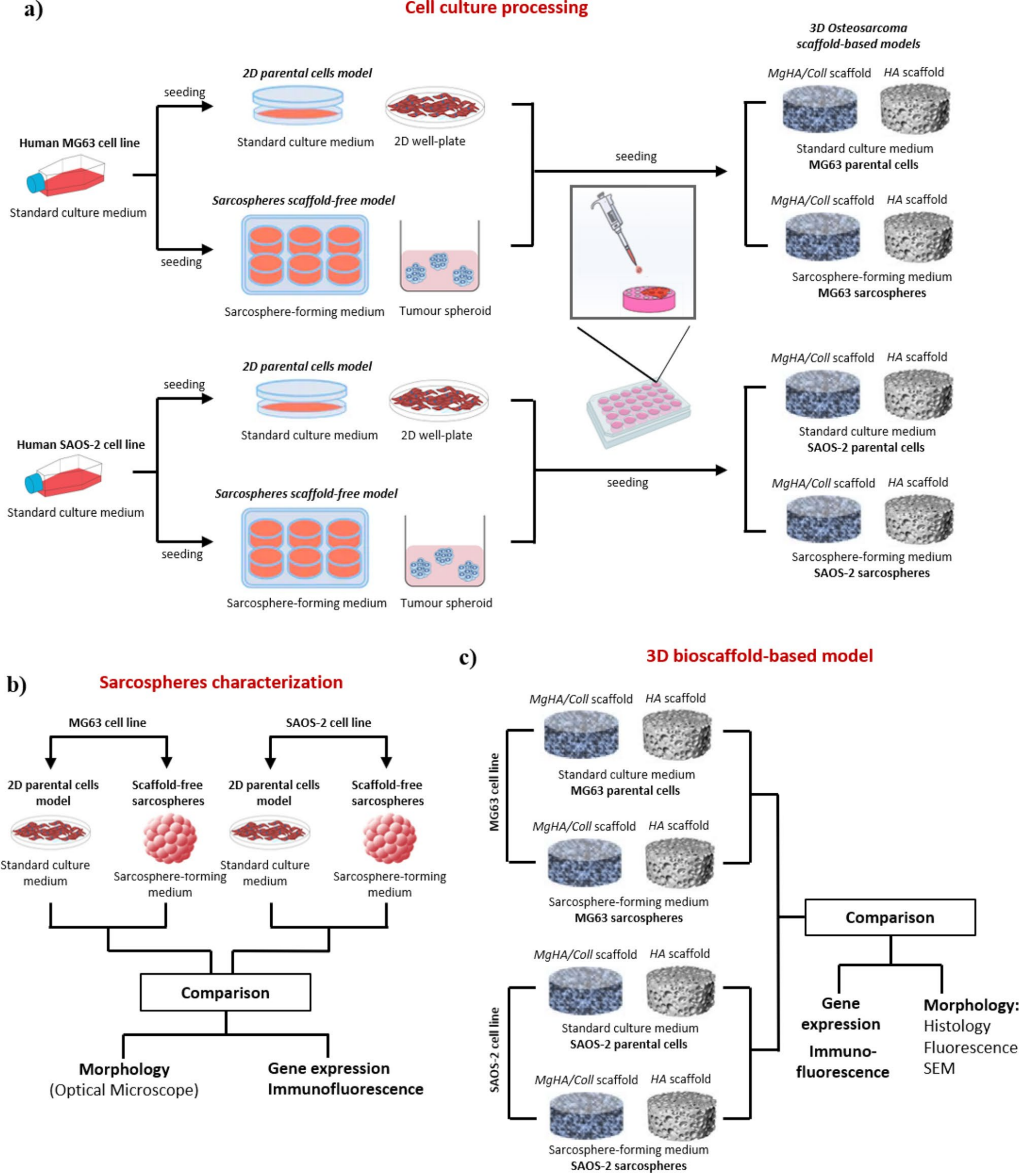
Graphical representation of the experimental plan. (**a**) Cell culture processing of tumour parental cells and sarcospheres of both cell lines for the development of the 3D scaffold-based models of osteosarcoma. (**b**) Sarcospheres characterization of both cell lines compared to 2D parental cells. (**c**) Experimental plan and characterization of the two phenotypes (parental and sarcospheres) in the 3D osteosarcoma models. Scaffold-free sarcospheres were used as a control.

The sarcospheres were obtained from parental SAOS-2 and MG63 cell lines cultured under specific culture conditions as reported in literature^35–37^. Both cell lines were seeded in Ultra-Low Attachment 6 well-plates (Corning Inc., NY) with a density of 20,000 cells/well in DMEM/F-12 GlutaMAX (Gibco) culture medium supplemented with a specific factors cocktail composed by 10 μl/ml N2 (Gibco), 20 μl/ml B27 (Gibco), 0.1 μl/ml FGF (Invitrogen) and 0.01 μl/ml EGF (PeproTech). The cocktail was added to each well every 2/3 days for a total of 10 days of culture at 37 °C and in 5% CO_2_ atmosphere conditions, observing sarcospheres formation. After their formation, the sarcospheres were mechanically dissociated by pipetting to facilitate cell counting and seeding in Ultra-Low Attachment 6-well plates with a density of 2.5 × 10^4^ cells/well for additional 10 days of culture, following the manufacturer’s instructions reported above. These new cultures were named sarcospheres scaffold-free models (Fig. 8a,b).

### The 3D osteosarcoma models

The 3D scaffold-based culture models were developed for parental cells and sarcospheres of both cell lines (Fig. 8a). The scaffolds were placed in a 24-well plate and pre-conditioned in complete culture medium for 24 h before cell seeding. The scaffolds were seeded with parental cells or sarcospheres with a density of 2.5 × 10^4^ cells/scaffold by carefully dropping 20 μL of cell suspension on material upper surface. After 30 min. incubation to allow cell pre-attachment, the specific culture media (1.5 ml/well) for the different cell cultures, were added to each well. The standard culture medium of parental cells was gently changed every 3 days and sarcospheres were supplied with fresh culture medium and factors cocktail every 3 days for a total of 10 days of culture. These culture systems were named 3D sarcospheres or parental cells scaffold-based models (Fig. 8a,c).

### Cell morphology analysis

#### Histological analysis

For histological analysis, the 3D MgHA/Coll models were washed in PBS 1X and fixed in 4% buffered formaldehyde for 15 min at room temperature. After washings in PBS 1X, the samples were placed in histological bio-cassettes and dehydrated with increasing scale alcohol passages (from 30 to 100% v/v), under vacuum conditions. Then, the samples were subjected to two final dehydration passages in xylene for 1 h under the same conditions. Samples embedding was performed by using liquid paraffin. A semi-automatic rotary microtome (Histo-Line Laboratories) was used to dissect the samples obtaining polyline slides series of 5 μm thick sections, which were hydrated with decreasing scale alcohol passages (from 100 to 30% v/v) before Haematoxylin–Eosin (H&E) staining. After staining, sections were mounted and visualized with an Optical Microscope (Nikon).

#### Actin and DAPI staining

The 3D MgHA/Coll and HA models were washed in PBS 1X (Gibco) for 5 min, then fixed in 4% (w/v) paraformaldehyde (PFA) (Sigma) for 15 min and permeabilized in PBS 1X with 0.1% (v/v) Triton X-100 for 5 min at room temperature. F-actin filaments were highlighted with FITC-conjugated fluorescein-phalloidin (Invitrogen, 38 mM) staining, followed by 4′-6-Diamidino-2-phenylindole (DAPI) (Invitrogen, 300 µM) counterstaining to identify cell nuclei, following the manufacturer’s instructions. The samples were visualized with an inverted Ti-E fluorescence microscope (Nikon).

#### Scanning electron microscopy (SEM) analysis

For SEM analysis, the 3D HA models were washed with 0.1 M Sodium Cacodylate Buffer pH 7.4 and fixed in 2.5% Glutaraldehyde in 0.1 M Sodium Cacodylate Buffer pH 7.4 for 2 h at 4 °C. After washing in 0.1 M Sodium Cacodylate Buffer pH 7.4, the samples were dehydrated with passages in a series of increasing scale alcohol, followed by two final passages with Hexamethyldisilazane (Sigma-Aldrich) at room temperature. Dehydrated samples were sputter-coated with gold (20 μm gold film) and observed by using Stereoscan 360 SEM (Cambridge Instruments, UK).

### Quantitative real time PCR (qPCR)

After 10 days of culture, the gene expression profile was analysed. The total RNA extraction and purification were performed by using the Tri Reagent and a purification kit (Direct-zol RNA MiniPrep kit, Zymo Research), following the manufacturer’s instructions. RNA quantification and purity degree were evaluated by using the NanoDrop One Microvolume UV–Vis Spectrophotometer (Thermo Scientific), following the manufacturer’s instructions. The single strand cDNA was produced by using the High-Capacity cDNA Reverse Transcription Kit (Applied Biosystem) starting from 500 ng of purified RNA, following the manufacturer’s instructions. The cDNA was subjected to Real-Time PCR by using the TaqMan Gene Expression Assay Kit (Applied Biosystem) to SOX-2 (Hs01053049_s1), NOTCH (Hs01062014_m1), Inteleukin 6 (Hs00174131_m1) and HIF-1α (Hs00153153_m1) were analysed in the 3D sarcospheres scaffold-based models with both HA and MgHA/Coll material by using scaffold-free sarcospheres as experimental control. GAPDH (HS99999905-M1) was used as housekeeping gene. Two different experiments with the same experimental plan were performed and three samples of each group were analysed using three technical replicates. Data were collected from StepOne Real-Time PCR System (Applied Biosystems) and the relative quantification of target gene was performed by using the comparative threshold (Ct) method (ΔΔCt) where relative gene expression level equals to 2^−ΔΔCt86^.

### Immunofluorescence analysis

The samples were fixed in 4% (w/v) paraformaldehyde, saturated with 1% Bovine Serum Albumin (PAA The Cell Culture Company) and 10% normal goat serum (Euroclone) for 30 min under slow agitation conditions. The samples were permeabilized with 0.3% (v/v) Triton X-100 for 20 min and incubated overnight at 4 °C with primary Anti-SOX-2 (1:200, MA1-014, Thermo Scientific), Anti-OCT-4/POU5F1 (2 µg/ml, Life Technologies). Secondary antibodies Alexa Fluor 488 goat anti-mouse (A11029, Molecular Probes) and Sheep Polyclonal anti-mouse (Ab50502, Abcam) were used for OCT-4 and SOX-2 detection, respectively, for 1 h in the dark at room temperature. Cell nuclei were stained with DAPI (300 nM). The images were acquired by an Inverted Ti-E fluorescence microscope (Nikon).

### Statistical analysis

Statistical analysis was performed by using GraphPad Prism software (version 6.0). The results are expressed as mean ± standard error of mean and they were analysed by two-way variance analysis (Two-way ANOVA) followed by Bonferroni's multiple comparisons test.
